# Quantification of DNA Damage in Different Tissues in Rats with Heart Failure

**DOI:** 10.36660/abc.20180198

**Published:** 2020-02

**Authors:** Giuseppe Potrick Stefani, Ramiro Barcos Nunes, Douglas Dalcin Rossato, Vitor Scotta Hentschke, Marlise Di Domenico, Pedro Dal Lago, Cláudia Ramos Rhoden

**Affiliations:** 1 Universidade Federal de Ciências da Saúde de Porto Alegre, Porto Alegre, RS - Brazil; 2 Centro Universitário Franciscano, Santa Maria, RS - Brazil

**Keywords:** Heart Failure, Rats, Rats Inbred Strains, Tissue Distribution, DNA Damage, Comet Assay

## Abstract

**Background:**

Chronic heart failure (CHF) is a complex syndrome which comprises structural and functional alterations in the heart in maintaining the adequate blood demand to all tissues. Few investigations sought to evaluate oxidative DNA damage in CHF.

**Objective:**

To quantify the DNA damage using the comet assay in left ventricle (LV), lungs, diaphragm, gastrocnemius and soleus in rats with CHF.

**Methods:**

Twelve male Wistar rats (300 to 330 g) were selected for the study: Sham (n = 6) and CHF (n = 6). The animals underwent myocardial infarction by the ligation of the left coronary artery. After six weeks, the animals were euthanized. It was performed a cell suspension of the tissues. The comet assay was performed to evaluate single and double strand breaks in DNA. Significance level (p) considered < 0.05.

**Results:**

The CHF group showed higher values of left ventricle end-diastolic pressure (LVEDP), pulmonary congestion, cardiac hypertrophy and lower values of maximal positive and negative derivatives of LV pressure, LV systolic pressure (p < 0.05). CHF group showed higher DNA damage (% tail DNA, tail moment and Olive tail moment) compared to Sham (p < 0.001). The tissue with the highest damage was the soleus, compared to LV and gastrocnemius in CHF group (p < 0.05).

**Conclusion:**

Our results indicates that the CHF affects all tissues, both centrally and peripherically, being more affected in skeletal muscle (soleus) and is positively correlated with LV dysfunction.

## Introduction

Heart failure is a complex syndrome which characterizes structural and functional abnormalities in the heart in maintaining adequate blood demand. Chronic heart failure (CHF) affects approximately 1 to 2% of the population in developed countries and its prevalence increases at least 10% in senior adults.^1^ One of the most common causes to heart failure is myocardial infarction (MI), which induces pathologic cardiac remodeling.^2^

This syndrome does not affect only the heart, it also affects other organs, such as lungs and skeletal muscles.^3^ CHF is characterized by changes in ventilatory mechanics which impair the uptake and supply of oxygen to the systems. Hypoperfusion, which is sustained with a ventricular dysfunction in a vicious cycle, induces oxidative stress in the majority of tissues.^4^ Oxidative stress is a state in which the cell is in an oxidative imbalance, forming more reactive species than its neutralizing capacity.^5^ It has been proposed elsewhere that oxidative stress biomarkers, such as concentration of malondialdehyde and uric acid, could enlighten the extent of oxidative damage and guide treatment in patients with CHF.^6^

Since reactive oxygen species (ROS) can damage different biomolecules, such as lipids, proteins and DNA, the damage in nucleic acids has not been consistently investigated in CHF. A biomarker that has already been a target of investigation is the concentration of 8-hydroxy-2'-deoxyguanosine (8-OHdG).^7^ However, its measurement mirrors the oxidative damage in one type of DNA lesion, which does not reflect the total damage in the DNA helix. For that, toxicological assays, such as the comet assay, have never been tested in CHF, aiming to assess global DNA damage in different tissues. This technique is broadly used in toxicological studies and is considered to be consistent, sensitive and highly reproductive.^8^

The comet assay directly measures the extent of DNA damage, constituted by single and double DNA strand breaks.^9^ This method allows its measurement in blood and all tissues of interest, expanding the analysis of local damage, and its correlation with physiological and functional parameters.^10^ In heart failure, it is still not clear how the inability of the failing heart can affect different structures beyond the cardiovascular system, especially on DNA damage. Since in CHF there is a scenario of systemic oxidative damage as a function of the chronicity of the syndrome, the objective of this study was to evaluate DNA damage in different tissues, such as left ventricle, lungs and skeletal muscles (diaphragm, gastrocnemius and soleus) in rats affected by the condition.

## Methods

### Animals

There was a selection of 12 male Wistar rats (100 days old, from 300 to 330 g) from the Animal Breeding Unit of *Universidade Federal de Ciências da Saúde de Porto Alegre* (UFCSPA, Brazil). The animals were housed in groups of three animals per cage, which received food and water *ad libitum* in an specific room maintained at 22°C under a 12:12-hour light-dark cycle.

The handling of the animals obeyed Law No. 11.794 of 10/08/2008, Law No. 6.899 of 07/15/2009, and Resolution Nº. 879 of 02/15/2008 (CFMV), as well as other provisions applicable to the use of animals for research. The experiment complied with resolutions of the National Council on Animal Experimentation, the Guide for the Care and Use of Laboratory Animals (Institute of Laboratory Animal Resources, National Academy of Sciences, Washington, D.C., 1996), as well as of the Ethical Principles in Animal Experimentation of the National Animal Experimentation Control Council (CONCEA). This study was approved by CEUA/UFCSPA, under protocol number 114/13.

### Induction of Myocardial Infarction (MI)

The animals were anesthetized with xylazine (12 mg/kg ip) and ketamine (90 mg/kg ip), intubated and artificially ventilated. The ligation of the left coronary artery was performed. Sham operations were performed as described elsewhere.^11^ After the surgeries, the animals received one injection of cetoprophane (5.4 mg/kg ip) every 6 hours - completing 48 hours - and penicillin (70,000 units/ml ip). The surgeries were performed by one surgeon. Post-surgery mortality rate was 15%. After MI induction, 6 weeks of recovery were designated, which was necessary for the animals to develop CHF. In order to document the animals that developed heart failure, we used different variables to characterize this syndrome, such as the presence of left atrial trombi, thoracic effusions, pulmonary congestion, left and right ventricular hypertrophy to body weight.^12^

### Heart Failure Condition

Those animals that presented left ventricular-end diastolic pressure (LVEDP) higher than 15.0 mmHg, as well as increased right ventricle weight to body weight ratio (> 0.8 mg/g) and the presence of pulmonary congestion were considered positive to CHF.^12-14^

### Hemodynamic Evaluation

After the sixth week, the animals were anesthetized with xylazine (12 mg/kg i.p.) and ketamine (90 mg/kg i.p.). A polyethylene catheter (PE-50) was placed into the right carotid artery. Arterial pressure was recorded and the catheter was positioned into the left ventricle to perform ventricular pressure recording. Data were registered by a pressure transducer (strain-gauge, Narco Biosystem Miniature Pulse Transducer RP-155, Houston, Texas, USA), coupled to a pressure amplifier. Pressure analogical signals were digitalized by a data acquisiton system (CODAS-Data Acquisition System, Akron, Ohio, USA) with a sampling rate of 2,000 Hz. These data were used to determine diastolic blood pressure (DBP), systolic blood pressure (SBP), mean blood pressure (MBP), heart rate (HR), left ventricular systolic pressure (LVSP), LVEDP and left ventricular maximum positive and negative dP/dt (+dP/dtmax, -dP/dtmax), as previously described.^15^ Animals that presented LVEDP higher than 15 mmHg in hemodynamic evaluation were considered with left ventricular dysfunction.^13^

### Tissue Collection

The animals were euthanized through intravenous infusion overdose of the anesthetic pentobarbital (80 mg/kg i.p.).^16^ After that, the lungs, the diaphragm, the right gastrocnemius, the right soleus and the heart were removed. The left ventricle was separated from the right one for the comet assay. All samples were stored at -80°C for posterior analysis.

### Determination of Infarct Size, Cardiac Hypertrophy and Pulmonary and Hepatic Congestion

The hearts were removed and weighted, without blood within the chamber and without atria. The size of the infarct area was determined by planimetry.^17^ To evaluate cardiac hypertrophy, organ mass was expressed as a proportion of body mass (tissue mass/body mass - mg/g).^18^ Animals with right ventricle hypertrophy (i.e. right ventricle mass-to-body weight ratio > 0.80 mg/g) were considered as rats that developed heart failure.^12^ To determine pulmonary and hepatic congestion, the lungs and liver of each animal were removed, weighted and dehydrated (80°C) for 48 hours, and then weighted again to evaluate water percentage.

### Single Cell Gel Electrophoresis (SCGE)

Single Cell Gel Electrophoresis (SCGE) was performed in alkaline conditions (pH > 13.0).^19^ All procedures were performed avoiding any direct incidence of light. For the assay, a cell suspension of the tissue (left ventricle, lungs, diaphragm, right gastrocnemius and right soleus) was primarily carried out in PBS buffer (pH = 7.40) with standard and gentle manual homogenization. This step required the observation of the density of cells that would be used in each slide. Neubauer’s chamber was used to count approximately 7.3 x 10^5^ cells/slide.

The suspension of cells (40 *µ*l) was added to agarose of low melting point (90 *µ*l). After gently mixed, this material was carefully superimposed over a slide previously covered with a thin agarose gel layer with a coverslip, and kept in a humid chamber at 4°C for 10 minutes, in order to further secure the suspension of tissue cells in the gel. Then, the coverslip was carefully removed and the slide was conditioned in a vertical cuvette containing lysis solution for at least 1 hour at 4°C.

The following step consisted in the unfolding of the cells, for 30 minutes in an alkaline buffer (pH > 10.0). Thereafter, it was followed by the process of electrophoresis, where lysed cells contained in the agarose gel were subjected to a voltage of 25 mV and 300 mA for 15 minutes in alkaline buffer solution (pH > 10.0). Then the plate was neutralized, stained with silver nitrate, rinsed and kept at room temperature to dry for later analysis. The slides of each animal were made in duplicate and a positive control of DNA damage with hydrogen peroxide (30 *µ*l/slide). The analysis was conducted under an optical microscope with a 20x increase by quantifying the size of the comet’s tail in 50 to 100 cells, according to the lengths, diameters, radii and dimensions of individual comets. Percentages of tail DNA, tail moment and Olive tail moment were used as damage quantification parameters.

### Quantification of DNA Damage

All the parameters presented in the results session regarding SCGE were calculated by the software CASP (CASP Labs®, Poland).^20^ The percentage of DNA in the tail, tail moment and Olive tail moment formulas are available for consultation in the supplementary data. Tail moment is characterized as the product of tail length and the percentage of DNA in the tail. The Olive tail moment, which is another parameter for DNA damage, comprises the product of the distance (relative to the x-axis) between the center of gravity of the head with the center of gravity of the tail of the comet and the percentage of tail DNA.

### Sample Size and Statistical Analysis

For a minimum difference of 23 arbitrary units of tail moment of ± 4 SD, it was possible to determine minimum statistical difference of two groups with three animals each.^21^ In our investigation, we decided to use six animals in each group. Data are presented in mean ± SD. The normality test of Shapiro-Wilk was used to assess variables distribution. For comparisons between groups, an unpaired Student’s t test and a two-way analysis of variance were performed among different tissues with a Tukey’s post hoc test. A significance of 5%was considered. Statistical analysis was carried out by using SigmaPlot, version 12.0 for Windows, and graphics were created by GraphPad Prism, version 5.0 for Windows.

## Results

### Morphological Parameters

The animals showed no difference regarding neither initial nor final body mass. Animals submitted to MI showed mean infarction area of 36%. It was possible to observe higher ratio of myocardial mass, right ventricle and left ventricle-to-body mass compared to sham group, indicating cardiac remodeling in both ventricles. Regarding the congestion in the lungs and liver, higher rates of the former were only observed in the CHF group (Table 1).

**Table 1:** Body mass, morphometric cardiac characteristics, infarcted area and pulmonary and hepatic congestion of sham-operated rats and rats with left ventricular dysfunction

Variables	Sham	CHF
Initial Body Mass (g)	330.25 ± 17.24	328.29 ± 18.12
Final Body Mass (g)	400.50 ± 29.61	356.38 ± 32.23
Infarcted Area (%)	---	36.39 ± 8.11
MM/BM (mg/g)	2.56 ± 0.08	3.29 ± 0.46*
LV/BM (mg/g)	1.88 ± 0.21	2.36 ± 0.47*
RV/BM (mg/g)	0.58 ± 0.12	1.40 ± 1.01*
Pulmonary Congestion (%)	65.67 ± 9.34	87.31 ± 3.36*
Hepatic Congestion (%)	70.46 ± 1.05	71.43 ± 1.07

Values are presented in mean ± SD; n = 6 for all groups. Sham, sham-operated rats; CHF: Chronic heart failure rats; MM/BM: Myocardial mass-to-body mass ratio; LV/BM: left ventricle mass-to-body mass ratio; RV/BM: right ventricle mass-to-body mass ratio.

* p < 0.05 compared to the Sham group.

### Hemodynamic Parameters

When compared to the sham group, lower mean blood pressure was observed in the CHF group. SBP and DBP, as well as HR showed no difference in relation to the control group (Table 2). Regarding ventricular pressure variables, LVSP and higher LVEDP was observed in the CHF group, when compared to the sham one (Table 2).

**Table 2:** Mean, diastolic and systolic blood pressure, left ventricle end diastolic pressure, left ventricle systolic pressure and left ventricular maximum/minimum change over time of sham-operated rats and rats with left ventricular dysfunction

Variables	Sham	CHF
MBP (mmHg)	93.01 ± 14.70	76.78 ± 5.83*
DBP (mmHg)	73.54 ± 16.28	67.54 ± 7.15
SBP (mmHg)	99.75 ± 20.91	85.93 ± 5.51
Heart Rate (bpm)	253.56 ± 70.84	245.19 ± 57.69
LVEDP (mmHg)	5.40 ± 2.26	32.55 ± 5.32*
LVSP (mmHg)	104.24 ± 6.03	89.15 ± 3.15*
+ dP/dt_max_ (mmHg/s)	6,264.33 ± 1,566.47	4,281.63 ± 708.75*
- dP/dt_max_ (mmHg/s)	5,209.63 ± 1,274.09	2,823.80 ± 540.65*

Values are presented in mean ± SD; n = 6 for all groups. Sham, sham-operated rats; CHF: Chronic heart failure rats; MBP: Mean blood pressure; DBP: diastolic blood pressure; SBP: systolic blood pressure; LVEDP: left ventricular end-diastolic pressure; LVSP: left ventricular systolic pressure; +dP/dt_max_: Maximal positive derivative of ventricular pressure; -dP/dt_max_: maximal negative derivative of ventricular pressure.

* p < 0.05 compared to the Sham group.

The maximal positive derivative of ventricular pressure (+dP/dt_max_) showed alterations in the CHF group, presenting lower values, as well as maximal negative derivative of ventricular pressure (-dP/dt_max_), which showed lower values when compared to the control group (Table 2).

### DNA Damage Parameters

**Figure 1:**
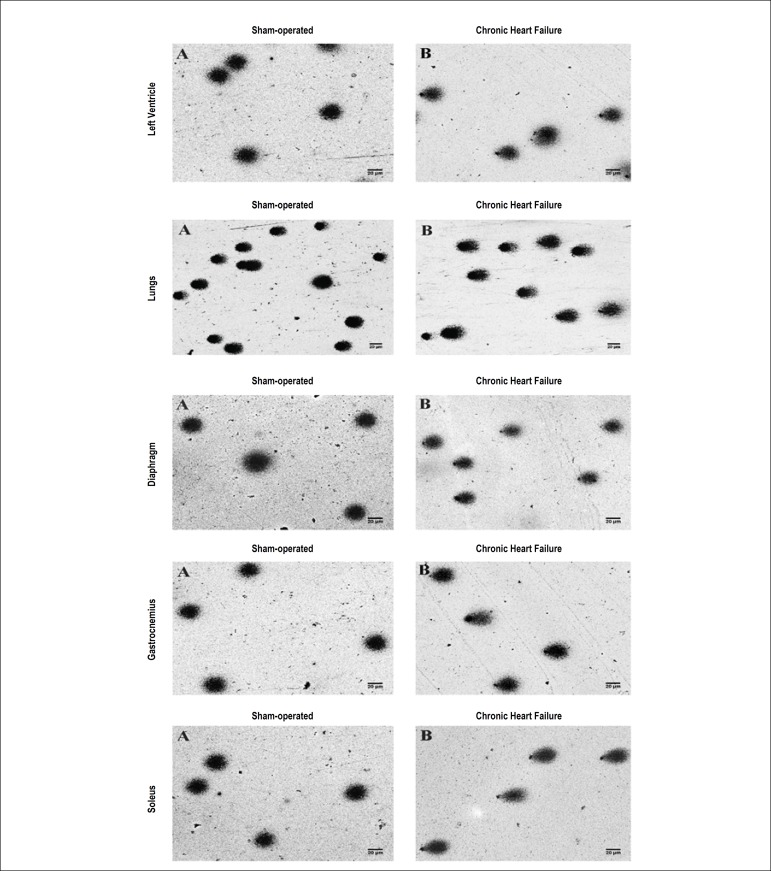
Image of cells submitted to SCGE (comet assay) of left ventricle, lungs, diaphragm, gastrocnemius and soleus of Sham-operated and rats with CHF. Panel A: Isolated cells of the respective tissue of Sham-operated rat. Panel B: Isolated cells of the respective tissue of rat with MI-induced CHF. Slides stained with silver nitrate. The image shows no formation of comets in Panel A, but in Panel B the image shows the formation of comets with distinct tails. The length of the tail represents the single strand and double strand breaks of nuclear DNA (magnification of 20x, scale bar of 20 µm).

Higher values of DNA damage were observed in all variables (% tail DNA, tail moment and Olive tail moment) in the CHF group, in all analyzed tissues (Table 3). DNA damage can be observed in the formation and frequency of comets in left ventricle, pulmonary, diaphragmatic, gastrocnemius and soleus cells (Figure 1).

**Table 3:** DNA quantification in different tissues of sham-operated animals and rats with chronic heart failure

		Sham			CHF	
	% Tail DNA	Tail Moment	Olive Tail Moment	% Tail DNA	Tail Moment	Olive Tail Moment
Left Ventricle	7.65 ± 3.35	0.77 ± 0.44	1.37 ± 0.59	33.29 ± 7.70*	10.51 ± 3.31*	7.04 ± 1.71*
Lungs	17.86 ± 3.93	6.76 ± 2.59	7.31 ± 2.15	36.20 ± 5.17*	23.30 ± 7.25*	19.10 ± 4.65*
Diaphragm	6.86 ± 2.63	1.40 ± 0.93	1.82 ± 0.79	41.23 ± 13.86*	14.06 ± 6.51*	9.82 ± 3.03*
Gastrocnemius	7.63 ± 4.66	1.04 ± 0.88	1.43 ± 0.70	28.07 ± 15.53*	8.69 ± 5.14*	6.17 ± 3.53*
Soleus	11.54 ± 2.46	1.53 ± 0.96	1.84 ± 0.76	55.79 ± 11.53*	20.90 ± 5.32*	12.83 ± 3.68*

Values are presented in mean ± SD; n = 6 for all groups. Sham, sham-operated rats; CHF: Chronic heart failure rats.

* = p < 0.01 versus Sham in relation to the variable and its corresponding tissue.

**Figure 2:**
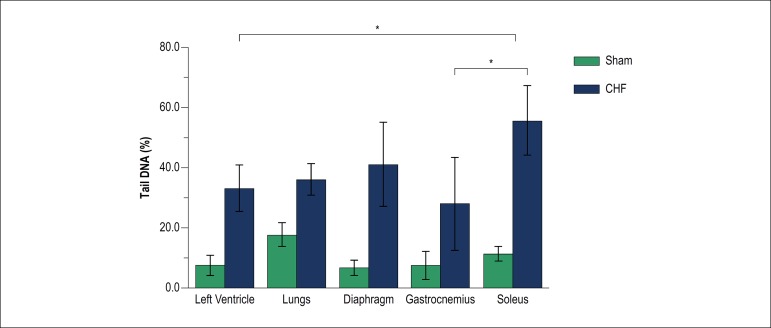
DNA damage in different tissues, according to % tail DNA, induced by CHF. Quantification of DNA damage in isolated cells of left ventricle, lungs, diaphragm, gastrocnemius and soleus muscles in Sham-operated rats and rats with CHF. Sham (n = 6), CHF (n = 6). Two-way ANOVA, with post-hoc test of Tukey. * p < 0.05 vs. Soleus.

Despite DNA damage being remarkably higher in CHF rats in all tissues when compared to other tissues in the same pathologic condition, it was observed higher damage in soleus compared to gastrocnemius and left ventricle in CHF group. The difference between tissue DNA damage in sham-operated animals and CHF ones can be observed in Figure 2.

## Discussion

Although there are some investigations using the comet assay in CHF, to the best of our knowledge, this is the first study to report the total extent of DNA damage in different tissues in an experimental model of CHF. The major finding of this investigation is the reproducibility and applicability of SCGE in the MI experimental model. Animals with CHF demonstrated higher extent of DNA damage than the control group in heart, lungs, diaphragm and skeletal muscles. This finding supports the main hypothesis that CHF affects the stability of DNA not locally, but systemically.

Since CHF is a complex syndrome, it is essential to investigate the extent of damage that the hypoperfusion may promote. We showed an *in vivo* model of CHF whose damage was ranging from two- to six-fold higher than in the absence of heart failure. The animals of this study demonstrated traditional alterations observable in the ligation of the left coronary artery model of heart failure in rats.^22,23^ Mean LVEDP above 30 mmHg was observed, which characterizes ventricular dysfunction.^14^ Also, traditional hemodynamic alterations were observed in animals with CHF, such as lower LVSP, and maximum positive and negative derivatives of ventricular pressure. Morphological parameters also showed meaningful alterations in left and right ventricle hypertrophy, as well as pulmonary congestion. All these parameters (hemodynamic and morphological) characterize the presence of CHF.^12,24,25^

The SCGE method performed in alkaline conditions allows the evaluation of global DNA damage. The damage observed in the comets is formed by single and double strand breaks that are unattached from the chromatin, in DNA fragments.^10^ The evaluation of 8-OHdG in patients with CHF has been recently proposed. The 8-OHdG is an oxidized purine base, one of the most frequent oxidative products of DNA.^26^ Most of the lesions in DNA may be manifested in single and double strand breaks, not only in oxidative by-products. Reactive oxygen species may damage DNA and form oxidative bases, such as 8-OHdG, 5-hydroxyuracil, 2-hydroxyadenine and 4,6-diamino-5-formamidopyridine.

Some caveats should be made before comparing results measured by nuclear DNA damage, as in our study, to results obtained in concentrations of oxidized purine base. The DNA damage measured by the comet assay reflects the overall damage, other than the 8-OHdG measurement that cannot assert the same.^27^

A recent meta-analysis demonstrated that eight studies evaluated the oxidative DNA damage to the specific DNA lesion of 8-OHdG. All investigations demonstrated higher concentrations of 8-OHdG in CHF patients.^28^ The rationale for higher concentrations of DNA oxidative products indicates that the higher extent of genotoxic damage is highly contributed to the oxidation of mitochondrial DNA.^29^ Cardiac myocytes present the highest content of mitochondria, which could indicate higher formation of ROS and contribute significantly to mitochondrial dysfunction. This study did not quantify the concentration of 8-OHdG, however we considered DNA damage that is widely used in order to evaluate DNA strand breaks. The study by Jaenisch et al.,^30^ also developed by our laboratory, used the same method of assessing DNA damage (alkaline version of the comet assay) in animals with CHF submitted to respiratory muscle training. Regarding the extent of DNA damage of Sham rats compared to rats with CHF, the percentage of DNA in the comet tail was relatively similar to the results obtained in the investigation on diaphragm cells.^30^

One interesting finding of this study was higher DNA damage in soleus cells than in left ventricle ones, which supports the fact that, after MI, the ventricle functionally and morphologically adapts and the peripheral muscle suffers histological and biochemical alterations.^31,32^ The acute phase of MI is characterized by the necrosis of cardiac myocytes, which expands the area of necrosis of the left ventricle in the following hours, affecting adjacent structures.^33^ In this phase of MI, the extent of DNA damage is probably higher than in any other tissue, as can be observed in pro-inflammatory cytokines and autophagic mediators.^34,35^

Since cardiomyocytes have a renewal rate of approximately 1% in young people, and about 0.45% in elderly,^36^ this fact reinforce our findings regarding the difference of DNA damage among the tissues of CHF rats. The left ventricle shows high adaptability to modify its geometry, and ability to repair major oxidative products of DNA. It has been demonstrated that the main problem of CHF is not the central alterations in the heart, but it also affects, indirectly, all other organs.^37^ The complexity of the CHF scenario, such as cardiac remodeling, changes in ventilatory mechanics and hemodynamics, and systemic pro-inflammatory state leads to the formation of free radicals of different ways. This critical dysfunctional status establishes an oxidative stress condition in different organs and systems.^38,39^

We hypothesized that skeletal muscle cells would have higher DNA damage in CHF.^40,41^ For this reason, we chose to analyze two different skeletal muscles (soleus and gastrocnemius muscles) by their different fiber type proportions in rats with CHF. The skeletal muscle in CHF is highly affected by the hypoperfusion which augments the oxidative damage within, especially in the mitochondria.^42^ Since the skeletal muscle is target of oxidative damage, it was expected to have higher damage observed in our findings. In CHF, the antioxidant defense system in skeletal muscles might be constantly decreased over time.^43^ The oxidative damage observed in the soleus muscle is very likely to be explained by its morphological characteristic (e.g. higher number of capillaries per fiber, predominance of type I fibers, greater activity of aerobic metabolism).^44,45^ On the other hand, the gastrocnemius muscle presents morphology of mixed characteristics with a more balanced percentage distribution in relation to the type of fibers; therefore, less dependence of aerobic metabolism. For this reason, we imagine that it presented less DNA damage than the soleus muscle.

Antioxidant machinery changes over time in the cell cycle (e.g.: myogenic proliferation and differentiation) demonstrating to have more expression of antioxidant enzymes activity in myoblasts than in myotubes, thus increasing the probability of mortality under oxidative stress.^5^ This phenomenon is interesting, since the disuse of skeletal muscles due to exercise intolerance is common in patients with CHF, in addition to being related to diminished antioxidant-stimulating trigger signaling of muscle contraction.^46^ Compared to the left ventricle, the soleus muscle does not have the same ability for adaptation, which may explain why the DNA damage was higher.

### Limitations

This work shows few limitations, such as the absence of DNA damage evaluation in other tissues (liver, encephalic structures and other skeletal muscles). Another limitation that may enrich our findings is the measurement of mutagenesis. Evaluating the mutagenesis of the CHF, along with the SCGE, might lead to a more robust scenario of DNA damage and its lack of repair of DNA lesions. Our design aims to evaluate the longitudinal damage that the experimental model of CHF might lead to and its differences regarding tissues; thus, the ability of DNA to repair its lesions could not be carried out.

## Conclusion

Our results show DNA damage using SCGE in the CHF experimental model by MI. The left ventricle dysfunction clearly affects the cardiac tissue, lungs, diaphragm, gastrocnemius and soleus and was associated with the extent of DNA damage, affecting the soleus muscle more than the left ventricle and the gastrocnemius. The comet assay was proven to be a reliable tool for quantifying DNA damage in different tissues of animals with CHF and the soleus muscle was shown to be more affected by the heart failure than the left ventricle and the gastrocnemius.
